# The Kissing Carotid Variant: Case Insights and Surgical Precautions in Anterior Cervical Discectomy and Fusion (ACDF)

**DOI:** 10.7759/cureus.91106

**Published:** 2025-08-27

**Authors:** Alyssa Burd, Aidan Gaertner, Leah Pavlish, Brian J Gill

**Affiliations:** 1 Orthopaedic Surgery, Creighton University School of Medicine, Omaha, USA; 2 Orthopaedic Surgery, Nebraska Spine and Pain Center, L.L.P., Omaha, USA

**Keywords:** acdf, anterior cervical discectomy and fusion (acdf), carotid artery anomaly, cervical spine surgery, kissing carotid arteries, preoperative planning

## Abstract

“Kissing” carotid arteries is a rare anatomical variant that describes the medialization of the internal and/or common carotid vasculature. While most patients remain asymptomatic, concerns for this anomaly arise during anterior surgical approaches.

A 73-year-old female patient presented to our orthopedic spine clinic with neck and radicular pain in her right hand in the C7 distribution. Cervical MRI revealed a right paracentral disc extrusion at C6-C7, causing moderate to severe spinal canal stenosis and right foraminal stenosis. Severe central stenosis was also noted at C4-C5, and moderate central stenosis with severe left foraminal stenosis at C3-C4. The common and internal carotid arteries were noted to have significant medialization along the anterior cervical spine beginning caudally at C7 and extending cephalad to the C4 vertebral body. Typically, an anterior cervical discectomy and fusion (ACDF) is preferred to address the underlying cervical pathology. Due to the anatomical variant potentially obfuscating the anterior cervical surgical approach, preoperative planning is critical. Additional imaging may be necessary to better clarify surgical planes. In this case, the patient eventually underwent a C4-C7 ACDF without intraoperative complications.

This case report highlights the potential risks and complications associated with aberrant carotid artery anatomy in patients undergoing ACDF. Although carotid injury in ACDF is rare, patients with aberrant carotids may be at increased risk of complications, such as postoperative stroke and direct incidental arteriotomy with excessive bleeding. While no immediate intraoperative complications occurred in this case, recognition of this variant and comprehensive surgical planning prior to surgery are crucial to mitigate potential intraoperative and postoperative morbidity.

## Introduction

The incidence of carotid artery medialization, or “kissing carotids,” is relatively rare, ranging from 2.6% to 10%, but it has important and life-threatening implications regarding anterior cervical surgical approaches [1,2]. In patients with symptomatic cervical disc herniations and radiculopathy, an anterior cervical discectomy and fusion (ACDF) is a well-accepted surgical approach if conservative management fails. This procedure involves decompression of the affected disc space, followed by interbody vertebral spacer stabilization with anterior plate and screw instrumentation [3]. The most common anterior cervical approach is the Smith-Robinson approach.

The most common complications of ACDF include postoperative dysphagia and laryngeal hoarseness [4]. Carotid artery injury is relatively rare but should not go unaddressed, specifically in cases of anatomical anomalies such as carotid artery medialization and aberrant vertebral arteries. The etiology of this anomaly has been proposed to be kinking and/or coiling of the carotids caused by congenital malformations, fibromuscular dysplasia, or age-related atherosclerotic changes [5]. While most patients with aberrant carotids remain asymptomatic, symptoms can include dysphagia, dysphonia, or a history of stroke and transient ischemic attack (TIA) [6]. The purpose of this case report is to highlight potential risks and complications associated with aberrant carotid artery anatomy in patients undergoing ACDF.

## Case presentation

**Figure 1:**
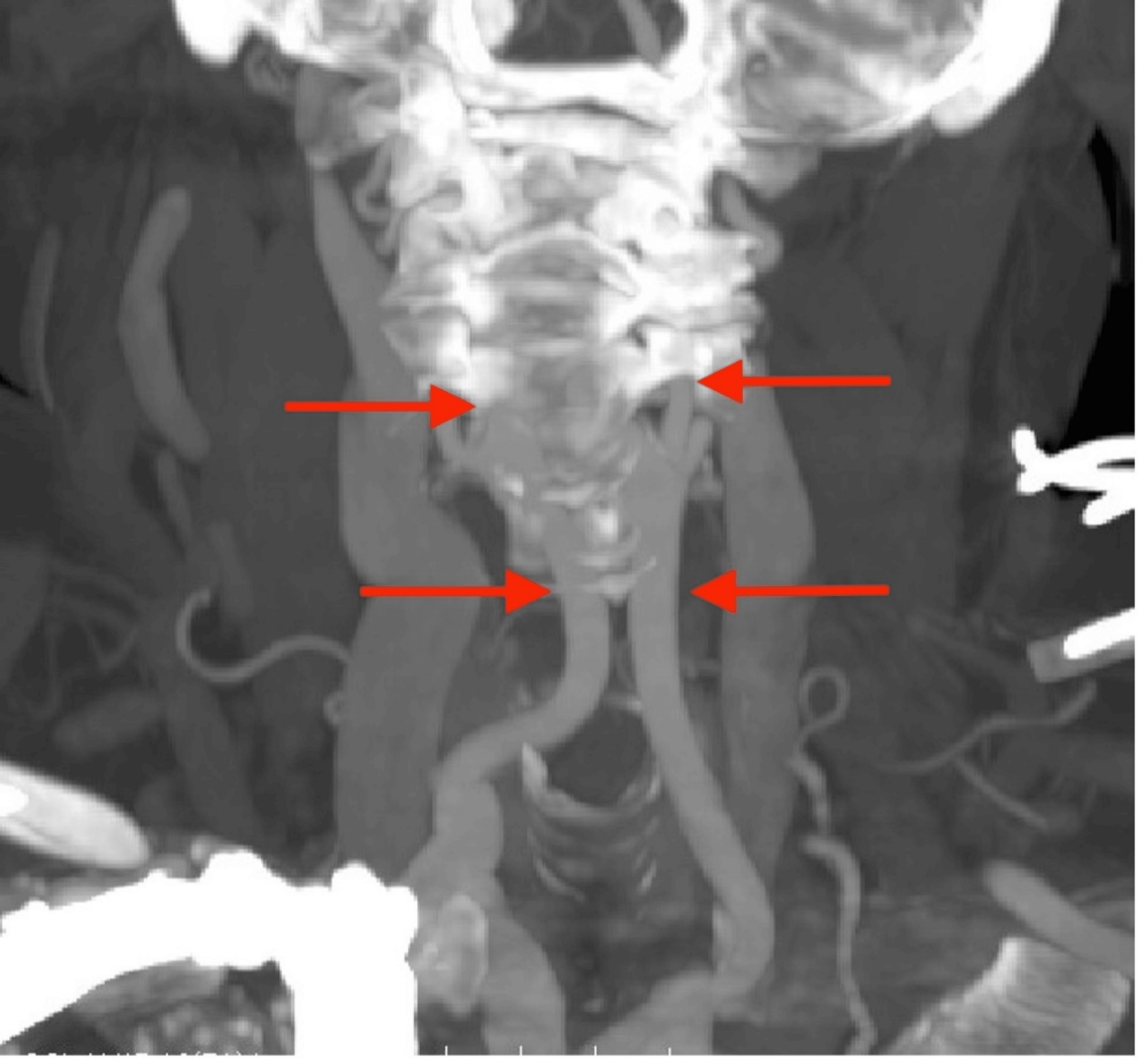
MRI of the neck demonstrates medialization of common carotids and overlying of internal carotids over the prevertebral spaces, indicated by the red arrows.

**Figure 2:**
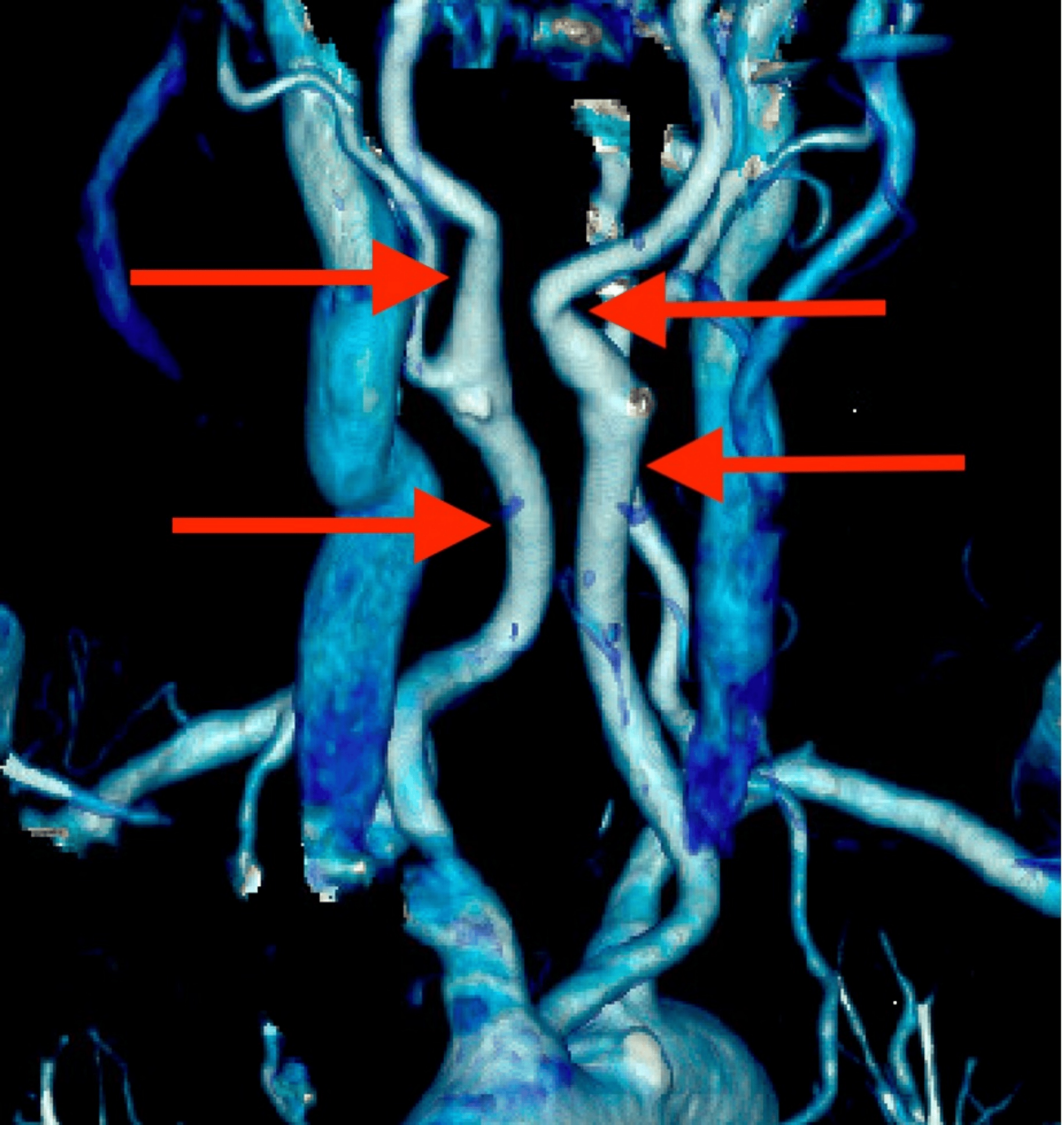
CT angiogram of the neck in coronal view demonstrating medialization of common and internal carotid arteries, indicated by the red arrows.

A 73-year-old female patient presented to the senior author’s clinic (JBG) with neck pain and paresthesia radiating to her right hand. She reported weakness in her right hand, as well as loss of balance and coordination. Her symptoms began three months before with no precipitating injury. Past pertinent medical and social history included a stroke and being a former smoker. Additional symptoms included dysphagia. On examination, decreased sensation was noted over the right radial forearm, thumb, and index finger, consistent with C6 radiculopathy. Right middle finger numbness was also noted, corresponding to C7 pathology. Strength and reflexes were normal. Cervical spine examination demonstrated a full range of motion with no pain upon movement. Spurling’s test was negative bilaterally. Cervical spine x-rays demonstrated cervical kyphosis from C2-C7 with normal disc height. A cervical MRI revealed a right paracentral disc extrusion at C6-C7 with both cephalad and caudal migration, causing moderate to severe spinal canal stenosis and right foraminal stenosis. Additionally, severe central stenosis was noted at C4-C5, and moderate central stenosis with severe left foraminal stenosis at C3-C4. Carotid architecture was not discussed in the initial MRI report. The treating physician (JBG) noted an aberrant right internal carotid artery positioned over the anterior vertebral bodies from C3-C7 (Figure 1). Radiology was contacted for a re-read and reported medial deviation of the common and internal carotid arteries along the cervical spine, starting at the level of C7 and extending to the level of C4. Further, a CT head and neck angiogram ordered prior to surgical consultation confirmed the medial carotid deviation (Figure 2). A posterior approach was discussed in lieu of ACDF with a cervical fusion from C2-T2 for correction and decompression to obviate the aberrant carotid anatomy. After much discussion, the patient proceeded with an ACDF from C4 to C7. No immediate operative complications were noted.

## Discussion

A cervical decompression and fusion is the recommended treatment option due to clinical symptoms of radiculopathy and confirmed imaging of disc protrusion and moderate to severe stenosis after failure of nonoperative care. Typically, an ACDF is performed to address the underlying pathology. Due to the aberrant carotid anatomy, a posterior cervical decompression and fusion (PCF) may be a more suitable surgical option to avoid potential carotid injury.

Prior to any surgical procedure, it is prudent to review pertinent anatomic structures, especially in patients with atypical findings, to avoid injury and surgical complications. Aberrant anatomical structures can be identified via MRI and CT angiogram imaging prior to surgery to help guide surgical planning. In the anterior cervical approach, the carotid sheath is an important landmark. Within the sheath lie the common and internal carotid arteries, the internal jugular vein, and the vagus nerve [7]. The carotid sheath normally can be identified by palpating lateral and posterior to the sternocleidomastoid muscle; this landmark can help prevent carotid artery injury. If there is difficulty palpating the sheath, sterile ultrasound or Doppler can be utilized intraoperatively for more definitive identification.

Regarding cervical levels, the common carotids bifurcate into the internal and external carotids at C4-5 [8]. Depending on which cervical levels are being addressed, this could be useful information for surgical planning.

The distribution of total cerebral blood flow is 72% through the internal carotid arteries [9]. Damage to these arteries can have detrimental effects on the brain, potentially resulting in stroke, especially in the absence of an intact Circle of Willis. In ACDF, cerebrovascular accident (CVA) is relatively uncommon, only occurring in 1:17,625 cases postoperatively [10]. During ACDF, the prolonged retraction of the carotid sheath can lead to plaque embolization, decreased arterial flow, intimal injury, or direct injury resulting in a stroke and/or excessive bleeding. Among patients who suffered a stroke post-ACDF, the majority were due to underlying carotid artery stenosis. Due to the protective sheath of the carotid artery, there is a low CVA incidence reported, less than 0.01%, for iatrogenic carotid artery injuries in ACDF. However, the true incidence of intraoperative carotid artery injury is unknown. The dominant theory for post-ACDF stroke is that retraction of the carotids causes decreased blood flow through the arteries, resulting in a 39% to 80% reduction in cross-sectional area [11]. Patients with aberrant carotids may be predisposed to a higher risk of stroke because the abnormal anatomy of the carotids may reduce arterial flow due to increased retraction pressure [12]. This potential complication should be taken into account by surgeons when considering anterior cervical approaches.

## Conclusions

While uncommon, the presence of aberrant carotid arteries should not be overlooked. It is critical to recognize these anomalies prior to anterior cervical approaches, such as ACDF, to obviate injury. Preoperative planning plays an important role in the success of cervical procedures. This can include performing a preoperative cervical CT scan or consulting with otolaryngology specialists regarding the aberrant anatomy. Additionally, if the surgeon is having trouble visualizing the carotid sheath, an ultrasound or Doppler of the neck can be performed for more conclusive evidence. In this patient, medialization of bilateral common and internal carotids was noted and assessed with additional imaging, a CT angiogram. When deciding on the best surgical approach for patients with aberrant carotids, the posterior approach may be considered.

In summary, “kissing carotids” is an uncommon arterial anomaly that, if not recognized preoperatively, may lead to potentially disastrous complications. While aberrant carotid arteries may not be seen in every surgeon’s practice, attention to detail through pre- and intraoperative planning prior to anterior surgical approaches can help prevent complications.
